# Effect of Combined Mycophenolate and Rapamycin Treatment on Kidney Fibrosis in Murine Lupus Nephritis

**DOI:** 10.3389/fphar.2022.866077

**Published:** 2022-04-28

**Authors:** Chenzhu Zhang, Tsz Wai Tam, Mel KM Chau, Cristina Alexandra García Córdoba, Susan Yung, Tak Mao Chan

**Affiliations:** Department of Medicine, The University of Hong Kong, Hong Kong, Hong Kong SAR, China

**Keywords:** lupus nephritis, rapamycin, mycophenolate, mesangial cells, fibrosis

## Abstract

**Background:** A significant proportion of lupus nephritis patients develop chronic kidney disease (CKD) and progressive kidney fibrosis, for which there is no specific treatment. We previously reported that mycophenolate or rapamycin monotherapy showed comparable efficacy in suppressing kidney fibrosis in a murine model of lupus nephritis through their direct action on mesangial cells. We extended our study to investigate the effect of combined mycophenolate and rapamycin treatment (MR) on kidney fibrosis in NZBWF1/J mice.

**Methods:** Female NZBWF1/J mice with active nephritis were randomized to receive vehicle or treatment with mycophenolate (50 mg/kg/day) and rapamycin (1.5 mg/kg/day) (MR) for up to 12 weeks, and the effect of treatment on clinical parameters, kidney histology, and fibrotic processes was investigated.

**Results:** Progression of nephritis in untreated mice was accompanied by mesangial proliferation, glomerulosclerosis, tubular atrophy, protein cast formation, increased mTOR and ERK phosphorylation, and induction of TGF-β1, IL-6, α-smooth muscle actin, fibronectin, and collagen expression. Combined MR treatment prolonged survival, improved kidney function, decreased anti-dsDNA antibody level, and ameliorated histopathological changes. The effect of combined MR treatment on kidney histology and function was comparable to that of mycophenolate or rapamycin monotherapy. *In vitro* studies in human mesangial cells showed that exogenous TGF-β1 and IL-6 both induced mTOR and ERK phosphorylation and downstream fibrotic processes. Both mycophenolic acid and rapamycin inhibited inflammatory and fibrotic processes induced by TGF-β1 or IL-6 by downregulating mTOR and ERK phosphorylation.

**Conclusions:** Our findings indicate that combined mycophenolate and rapamycin, at reduced dose, improves kidney fibrosis in murine lupus nephritis through their distinct effect on mTOR and ERK signaling in mesangial cells.

## Introduction

Lupus nephritis is a severe and common manifestation of systemic lupus erythematosus (SLE), a debilitating autoimmune disease characterized by loss of self-tolerance, autoantibody production, aberrant activation of both innate and adaptive immune responses, and immune-mediated kidney injury. Treatment of lupus nephritis is challenging because clinical presentation and response to treatment and prognosis vary considerably between patients and are influenced by genetics, gender, ethnicity, time of presentation, renal reserve, adherence to treatment, and pharmacogenomics. The current standard-of-care treatment for lupus nephritis necessitates high-dose glucocorticoids administered with mycophenolate or cyclophosphamide to induce remission, followed by long-term maintenance with low-dose glucocorticoids and either mycophenolate or azathioprine to prevent relapse (Chan et al., 2005; Mok et al., 2014; Chan, 2015; Yap et al., 2017). Chronic kidney disease (CKD) is prevalent in patients with lupus nephritis due to nephron loss associated with each episode of nephritis flare (Mageau et al., 2019). End-stage renal disease (ESRD) from progressive CKD is the main contributor to inferior patient survival (Yap et al., 2012b; Rijnink et al., 2017; Parikh et al., 2020). The side effects of immunosuppressive agents and inability of some patients to tolerate the target dose of immunosuppressive agents also contribute to failure to induce disease quiescence. There is, therefore, a need to develop new treatment strategies to prevent CKD and preserve long-term kidney and patient survival (Yap et al., 2012b).

CKD is characterized by the accumulation of matrix proteins in the kidney parenchyma, resulting in glomerulosclerosis and interstitial fibrosis (Duffield, 2014). There is currently no treatment for kidney fibrosis. Mycophenolate inhibits lymphocyte proliferation through its effect on inosine monophosphate dehydrogenase (Allison and Eugui, 2000). Emerging evidence suggests that the antiproliferative properties of mycophenolate extend beyond lymphocytes to non-immune cells including mesangial cells, proximal tubular epithelial cells, and fibroblasts, and mycophenolate can also reduce fibrotic processes in these cells (Badid et al., 2000; Dubus et al., 2002; Azzola et al., 2004; Copeland et al., 2007; Yung et al., 2009; Yung et al., 2015a; Yung et al., 2017; Zhang et al., 2019). Dysregulation of the mammalian or mechanistic target of rapamycin (mTOR) signaling pathway is observed in patients and mice with active lupus nephritis, and mTOR activation contributes to inflammatory and fibrotic processes (Warner et al., 1994; Lui et al., 2008a; Lui et al., 2008b; Zhang et al., 2019).

We previously investigated the effect of mTOR inhibitor rapamycin and also mycophenolate on kidney fibrosis in lupus-prone mice and demonstrated comparable efficacy of each in improving renal histopathology including fibrosis and kidney function. Our findings provided original evidence of the antifibrotic effects of monotherapy with mycophenolate or rapamycin in murine lupus nephritis, mediated through their direct actions on mesangial cells (Zhang et al., 2019). Additional potential clinical benefits of mTOR inhibitors include their antiviral effect and decreased incidence of malignancies (Campistol et al., 2006; Tedesco-Silva et al., 2015; Yanik et al., 2015). Studies have shown that SLE patients have an increased incidence of malignancies, attributed to both immune dysregulation and prior exposure to toxic immunosuppressive agents such as cyclophosphamide (Bernatsky et al., 2013; Ladouceur et al., 2018). Therefore, mTOR inhibitors may have a role in the clinical management of lupus nephritis patients, especially in those who cannot tolerate standard-of-care treatments (Chan et al., 2021).

Combination therapy is frequently used in the management of immune-mediated kidney diseases aiming to achieve efficacy, while reducing toxicity associated with individual drugs (Woo et al., 1995). Kidney transplant recipients treated with triple immunosuppression comprising mycophenolate, rapamycin, and corticosteroid showed markedly reduced gene expression of inflammatory and fibrosis mediators and reduced progression rate of chronic allograft nephropathy with better preservation of kidney structure and function compared to patients treated with calcineurin inhibitors and corticosteroids (Flechner et al., 2004). In this study, we investigated the effect of combined mycophenolate and rapamycin at reduced doses compared with that used in monotherapy on kidney fibrosis in active murine lupus nephritis.

## Materials and Methods

### Chemicals, Assays, and Drugs

All chemicals were of the highest purity and were purchased from Sigma Aldrich (Tin Hang Technology Ltd., Hong Kong), unless otherwise stated. QuantiChrom™ Creatinine, Urea and Albumin Assay Kits were purchased from BioAssay Systems, California, United States. Mouse Anti-dsDNA Antibody Quantitative ELISA Kits were purchased from Alpha Diagnostic Inc. (Onwon Trading Ltd., Hong Kong). Primary human mesangial cells (HMCs) were purchased from Lonza Cologne GmbH (Gene Company Limited, Hong Kong). Tissue culture flasks were purchased from Falcon (Becton-Dickenson, Gene Company Limited, Hong Kong). RPMI 1640 culture medium, fetal bovine serum (FBS), l-glutamine, and penicillin/streptomycin were purchased from Life Technologies Ltd. (Thermo Fisher Scientific, Hong Kong). Mouse anti-human fibronectin antibody (clone IST-4), rabbit anti-fibronectin antibody (product no. SAB5700724), mouse anti-human β-actin antibody (clone AC-74), HRP-conjugated rabbit anti-goat IgG antibody (product no. A8919), and FITC-conjugated anti-mouse IgG antibody (product no. AP127F) were purchased from Sigma Aldrich (Tin Hang Technology Ltd., Hong Kong). Rabbit anti-α-smooth muscle actin antibody (product no. ab5694) and rabbit IgG isotype control (product no. ab125938) were purchased from Abcam, Hong Kong). Goat anti-type I collagen (product no. 1310-01) and anti-type III collagen (product no. 1330-01) were purchased from SouthernBiotech (Genetimes Technology International Holding Limited, Hong Kong). Antibodies to TGF-β1 (clone 3C11) and IL-6 (clone M-19) were purchased from Santa Cruz Biotechnology Inc. (Genetimes Technology International Holding Limited, Hong Kong). Antibodies to phosphorylated (phospho) (Ser^2448^ and Ser^2481^; product nos. 2971 and 2974, respectively) and total mTOR (product no. 2972), phospho and total AKT (product nos. 9271 and 9272, respectively), phospho and total ERK (product nos. 4370 and 9102, respectively), and HRP-conjugated goat anti-rabbit IgG antibody (product no. 7074) were purchased from Cell Signaling Technology (Gene Company Limited, Hong Kong). Alexa Fluor 488 goat anti-rabbit IgG antibody (product no. A11070), Texas Red-X–conjugated goat anti-rabbit IgG antibody (product no. T6391), HRP-conjugated goat anti-human IgG antibody (product no. 31410), HRP-conjugated goat anti-mouse IgG antibody (product no. A16066), and goat IgG isotype control (product no. 31245) were purchased from ThermoFisher Scientific, Hong Kong. Recombinant human IL-6 and TGF-β1 were purchased from R&D Systems (Gene Company, Hong Kong). The BD OptEIA human IL-6 ELISA kit was purchased from BD Biosciences Pharmigen (Bio-Gene Technology Limited, Hong Kong). Mycophenolate was provided by Roche Diagnostics (Palo Alto, California, United States), and rapamycin (Rapamune) was purchased from Wyeth Hong Kong Limited (animal studies) and provided by Pfizer (New York, United States) (*in vitro* studies).

### Animal studies

All animal studies were approved by the Institutional Committee on the Use of Live Animals in Teaching and Research. Female NZBWF1/J mice were purchased from the Jackson Laboratory (Bar Harbor, Maine, United States) and were housed in a specific pathogen-free animal facility at the University of Hong Kong. The mice were placed under normal housing conditions in a 12-h night and day cycle. Water and standard chow were available *ad libitum*. Treatment commenced when mice were 23–25 weeks of age when they developed proteinuria, defined as >300 mg/dl detected on two separate occasions at least 2 days apart. The mice were randomized into four groups to receive vehicle (control group), monotherapy with mycophenolate (100 mg/kg/day) (M group) or rapamycin (3 mg/kg/day) (R group), or combined mycophenolate (50 mg/kg/day) and rapamycin (1.5 mg/kg/day) (MR group) for periods up to 12 weeks. In the MR group, the doses represent the lowest dose of each drug that could be used in combination to reduce proteinuria and collagen expression determined by Masson’s trichrome staining after 6–12 weeks of treatment. Treatment was administered by daily oral gavage for 6 and 12 weeks, following which the mice were killed, blood collected and kidneys harvested. Twenty-four hour urine sample was collected prior to sacrifice by placing mice in metabolic cages. After 12 weeks of treatment, some mice had follow-up six or 12 weeks post therapy to determine treatment sustainability (n = 6 mice per treatment per time-point). Six mice with established proteinuria as defined above were killed at the start of the study to obtain baseline clinical, serologic, and histological data (T = 0).

### Assays

All samples were measured in duplicate for all assays. Serum creatinine and urea levels were measured using QuantiChrom™ Creatinine and Urea Assay Kits, respectively. Spot urine was collected weekly, and the albumin-to-creatinine ratio (ACR) was determined using QuantiChrom™ Albumin and Creatinine Assay Kits to assess disease progression. Twenty-four hour urine sample was collected prior to sacrifice and albuminuria assessed. The anti-dsDNA antibody level was determined in serum samples using anti-dsDNA IgG quantitative ELISA kits according to the manufacturer’s instructions. Lower and upper limits of detection were 50 IU/ml and 1,000 IU/ml, respectively, and values greater than mean +2 SD of the anti-dsDNA antibody level detected in 36-week-old C57BL/6N mice, that is, 15.84 IU/ml [12.04 + (2 × 1.90)] were considered seropositive.

### White Blood Cell Count

The number of circulating white blood cells in vehicle and treated mice at the time of sacrifice was assessed by two independent observers without knowledge of the treatment and expressed as number of cells/ml (Yung et al., 2015b).

### Renal Histopathology

Paraffin-embedded kidney sections (5 μm) from untreated and treated mice were stained with H&E and Masson’s trichome as previously described (Zhang et al., 2019). Renal histology and collagen deposition were scored by two independent observers in a blinded manner. Briefly, kidney lesions relating to inflammation and fibrosis in the glomerular and tubulo-interstitial compartments were graded 0 to 3 (0 = normal, 1 = mild, 2 = moderate, and 3 = severe) and expressed as mean glomerular and tubulo-interstitial lesion scores for each group (Zhang et al., 2019). For each mouse, approximately 20 glomeruli, tubular, interstitial, and vascular areas were evaluated for glomerular hypercellularity, mesangial matrix expansion, crescent formation, influx of mononuclear cells, fibrinoid necrosis, hyaline deposits, tubular atrophy, protein cast deposition, and vasculopathy (Moreth et al., 2010; Yung et al., 2015b). Glomerular tuft area was assessed using Axiovision software. For semi-quantitative assessment of Masson’s trichrome staining, the images of approximately 15 glomeruli and tubules per mouse kidney were captured and graded as follows: 0 = 0–5% staining; 1 = 6–25% staining; 2 = 26–50% staining; 3 = 51–75% staining; 4 = >75% staining (Janssen et al., 2003).

### Cytochemical and Immunohistochemical Staining

Paraffin-embedded kidney sections (8 μm) from untreated and treated mice were stained for IL-6, TGF-β1, α-smooth muscle actin, fibronectin, and collagen I and collagen III, as previously described (Yung et al., 2009; Yung et al., 2015b; Zhang et al., 2019). Signal detection and visualization was performed by the peroxidase-anti-peroxidase method, and specimens were counterstained with hematoxylin (Yung et al., 2009). Staining of fibrotic mediators in the capillary loops, mesangium, and tubulo-interstitium was assessed semi-quantitatively in a blinded manner in approximately 15 glomeruli and tubules per mouse kidney and graded as previously described (Janssen et al., 2003). IgG deposition and phosphorylated mTOR and ERK were assessed in frozen renal sections (8 μm) from control and MR-treated mice using indirect immunofluorescence staining (Zhang et al., 2019). Briefly, to assess mTOR and ERK phosphorylation, sections were incubated with phospho-mTOR or anti–phospho-ERK antibodies followed by Texas Red or Alexa Fluor 488 conjugated goat anti-rabbit secondary antibodies, respectively. To determine IgG deposition, the kidney sections were incubated with FITC-conjugated anti-mouse IgG. The sections were mounted in a fluorescent mountant and epifluorescence viewed using a Nikon 80i upright fluorescent microscope and Spot RT3 slider digital camera system (Chintek Scientific (China) Ltd., Hong Kong). Fifteen glomeruli per kidney section were analyzed, and fluorescent staining in the glomerular capillary walls and mesangium was scored blindly on a scale of 0–3 (0 = no staining; 1 = weak staining; 2 = moderate staining, 3 = strong staining) (Herber et al., 2007; Yung et al., 2010).

### Human Mesangial Cells

Primary HMCs were maintained in RPMI 1640 medium supplemented with l-glutamine (2 μM), penicillin (100 U/ml), streptomycin (100 μg/ml), insulin (5 μg/ml), transferrin (5 μg/ml), and 10% FBS. All experiments were performed on the cells of the fifth to seventh passage that has been growth-arrested for 48 h. To identify signaling pathways and matrix proteins induced by IL-6 and TGF-β1, HMCs were pre-incubated with inhibitors to mTOR (rapamycin, 3 ng/ml), ERK (PD98059, 10 μM) and PI3K (LY294002, 25 μM), or rapamycin in combination with either PD98059 or LY294002 for 1 h at 37°C prior to incubation with serum-free medium (SFM), IL-6, or TGF-β1 (10 ng/ml final concentration, for both) for 24 h, after which time the supernatants were collected and the cells washed with PBS and lysed with 20 mM sodium acetate, pH 6.0, containing 4 M urea, 1% Triton X-100, and a cocktail of proteinase inhibitors (200 μl) (Yung et al., 2010; Yung et al., 2015a). We have chosen to stimulate HMCs with IL-6 and TGF-β1 since renal expression of both is increased in patients, and mice with active lupus nephritis and both drive tissue fibrosis (Yamamoto et al., 1996; Fielding et al., 2014). To determine the effect of mycophenolic acid (MPA) or rapamycin on inflammatory and fibrotic processes, HMCs were incubated with MPA (1 and 5 μg/ml) or rapamycin (1 and 3 ng/ml) for 1 h at 37°C followed by incubation with SFM, IL-6, or TGF-β1 (10 ng/ml, for both) for periods up to 72 h and samples processed as described above. The concentrations of MPA and rapamycin used in these studies represent blood trough levels in lupus nephritis patients and renal transplant recipients when given a daily dose of 2–3 g mycophenolate and 2–5 mg rapamycin, respectively (Hauser et al., 1999; Kahan et al., 2000; Borrows et al., 2006; Yap et al., 2018; Yap et al., 2020). The supernatants were used to determine the effect of MPA and rapamycin on IL-6 secretion, and cell lysates were used to assess phosphorylation of signaling pathways, α-smooth muscle actin, and matrix protein expression.

### Measurement of IL-6 in the Culture Supernatant

HMCs were incubated with SFM or TGF-β1 in the presence or absence of MPA or rapamycin for up to 72 h, after which time the supernatant was collected and centrifuged at 3,000 rpm for 10 min to remove any cell debris. Secreted IL-6 was determined using the IL-6 OptEIA™ ELISA kit according to the manufacturer’s instructions. Lower and upper limits of detection were 5 pg/ml and 300 pg/ml, respectively. All samples were measured in duplicate in serial dilution and normalized to their cellular protein content.

### Western Blot Analysis

Aliquots of cell lysates (20 μg total protein content) were separated under denaturing conditions on 8% polyacrylamide gels to determine fibronectin and collagen III expression and on 10% polyacrylamide gels to determine the expression of phospho- and total mTOR, phospho- and total ERK, phospho- and total AKT, α-smooth muscle actin, and β-actin. The samples were transferred onto nitrocellulose membranes and incubated with the relevant primary antibodies followed by the addition of secondary antibodies as previously described (Yung et al., 2009; Yung et al., 2010; Zhang et al., 2019). The bands were visualized with ECL, semi-quantitated by densitometry using ImageJ (NIH, United States), and expressed as arbitrary densitometric units (DU). Phospho-mTOR, phospho-ERK, and phospho-AKT were normalized to total mTOR, ERK, and AKT, respectively, and α-smooth muscle actin, fibronectin, and collagen III expression were normalized to β-actin.

### Statistical Analyses

The results from our animal studies were expressed as mean ± SEM (n = six to eight for each time-point per group). All *in vitro* studies were repeated at least three times and data expressed as mean ± SD. Statistical analysis was performed using Prism 6.0 for Windows (GraphPad Software, Inc., California, United States). Mouse survival was determined using Fisher’s exact test. The D’Agostino–Pearson normality test was used to assess normal distribution. Repeated measures ANOVA followed by Bonferroni’s multiple comparison post-test was used to assess intragroup and intergroup comparisons with three groups or more. Ordinary ANOVA followed by Bonferroni’s multiple comparison post-test was used to assess intergroup comparison for *in vitro* studies. Two-tailed *p* < 0.05 was considered statistically significant.

## Results

### Effect of Combined Mycophenolate and Rapamycin on Survival Rate, Kidney Function, and anti-dsDNA Antibody Titer in NZBWF1/J Mice

After 12 weeks of treatment, peripheral blood lymphocyte count in M, R, and MR groups was significantly lower than that in untreated mice (12.60 ± 0.81, 6.73 ± 0.35, 6.00 ± 0.72 and 6.20 ± 1.01 × 10^6^ lymphocyte count per ml blood for vehicle, M, R, and MR groups, respectively, *p* < 0.01, Vehicle vs. M, R, or MR). The immunosuppressive actions of M, R, and combined MR were comparable between all treated groups. None of the mice in the treatment groups developed infection, and body weight did not differ between the groups throughout the course of the study (data not shown).

**FIGURE 1:**
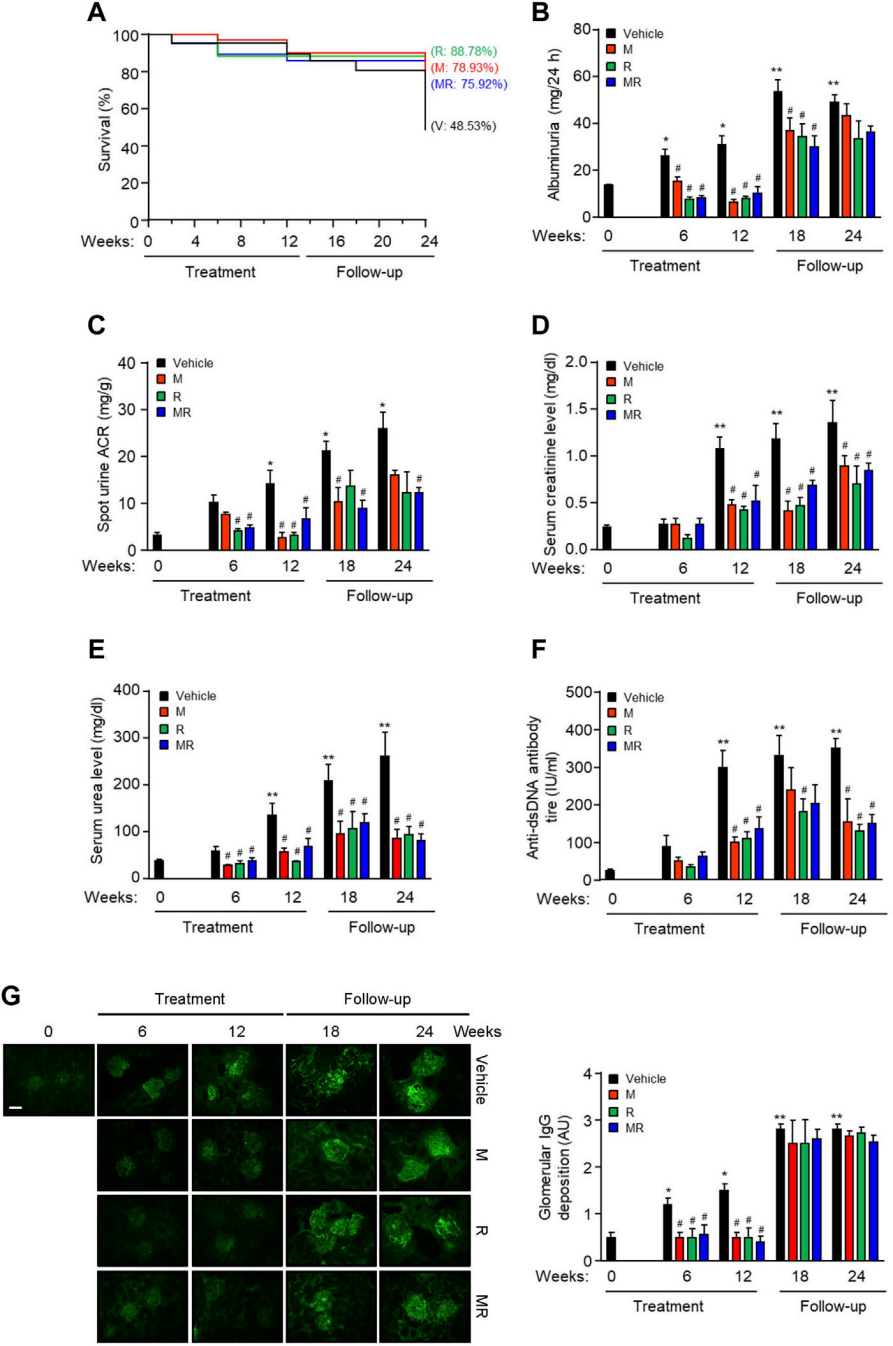
Effect of combined mycophenolate and rapamycin on survival, clinical, and serological parameters and IgG deposition in NZBWF1/J mice. The effect of vehicle (V), monotherapy mycophenolate (M) or rapamycin (R), or combined mycophenolate and rapamycin (MR) on **(A)** survival curves, **(B)** albuminuria, **(C)** spot urine albumin-to-creatinine ratio (ACR), **(D)** serum creatinine level, **(E)** serum urea level, and **(F)** circulating anti-dsDNA antibody titer in NZBWF1/J mice. **(G)** Representative images showing IgG deposition in vehicle-, M-, R-, and combined MR-treated mice at baseline (T = 0), 6, 12, 18, and 24 weeks. Original magnification x 200; Scale bar: 20 µm. Glomerular IgG deposition was graded as described in the *Materials and Methods* and data expressed as mean ± SEM (right panels). AU, arbitrary units. Data expressed as mean ± SEM (n = 6 mice per time-point per group). Data analyzed using Fisher’s exact test for panel A and repeated measures ANOVA with Bonferroni’s multiple comparison post-test for panels B–G. *p < 0.05, **p < 0.01, compared to baseline; ^#^ *p* < 0.05, compared to vehicle for the same time-point.

The survival rate for vehicle-, M-, R-, and MR-treated mice was similar after 12 weeks treatment (*P*=NS). Twelve weeks after cessation of treatment, the survival rate was 48.53, 78.93, 88.78, and 75.92%, respectively (*p* < 0.05, vehicle vs. M, R, or MR). There was no statistical difference in the survival rate for M-, R-, and MR-treated mice (Figure 1A). Albuminuria and serum creatinine, urea, and anti-dsDNA antibody levels increased with progressive nephritis in untreated mice, whereas M, R, and MR treatment significantly reduced these clinical and serological parameters of disease after 6–12 weeks and was sustained for 12 weeks after cessation of treatment (Figure 1B–F).

### Effect of Combined Mycophenolate and Rapamycin on IgG Deposition and Kidney Histology

**FIGURE 2:**
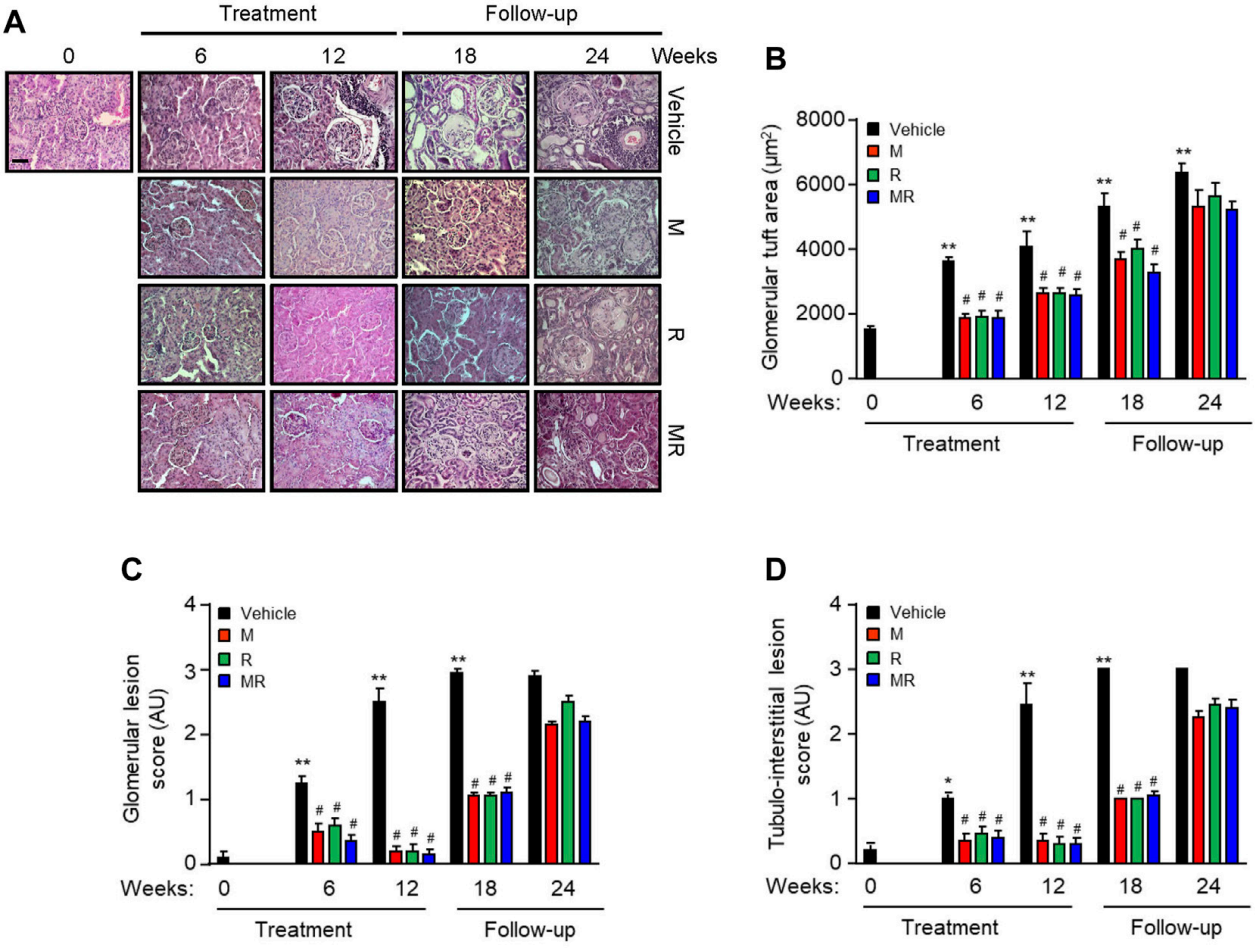
Effect of combined mycophenolate and rapamycin on kidney histology in NZBWF1/J mice **(A)** Representative images showing renal histopathology in vehicle-, M-, R-, and MR-treated mice as determined by H&E staining at baseline, 6, 12, 18, and 24 weeks. Original magnification x 200; Scale bar: 20 µm. **(B)** Glomerular tuft area, **(C)** glomerular lesion score, and **(D)** tubulo-interstitial lesion score for each group were graded as described in the *Materials and Methods*, and mean scores ±SEM are shown (n = 6 mice per time-point per group). AU, arbitrary units. Data analyzed using repeated measures ANOVA with Bonferroni’s multiple comparison post-test. *p < 0.05, **p < 0.01, compared to baseline; ^#^ *p* < 0.05, vehicle vs. treated groups for the same time-point.

Glomerular IgG deposition increased with progressive nephritis, whereas treatment with M, R, or MR significantly reduced IgG deposition after 6 weeks of treatment and was sustained for 12 weeks. IgG deposition increased in M, R, and combined MR groups once treatment was stopped (Figure 1G). Progression of nephritis in untreated mice was accompanied by mesangial expansion, glomerular hypertrophy, and infiltration of inflammatory cells in the periglomerular and tubulo-interstitial compartments (Figure 2). As disease progressed, tubular atrophy, cast formation, glomerulosclerosis, and interstitial fibrosis were detected 18–24 weeks after commencement of the study. M, R, and MR treatment attenuated histopathological changes after 12 weeks, but these abnormalities appeared 12 weeks after cessation of treatment (Figure 2).

### Effect of Combined Mycophenolate and Rapamycin on Glomerular mTOR and ERK Phosphorylation, and Expression of TGF-β1, IL-6, α-smooth Muscle Actin, Fibronectin, and Collagen

**FIGURE 3:**
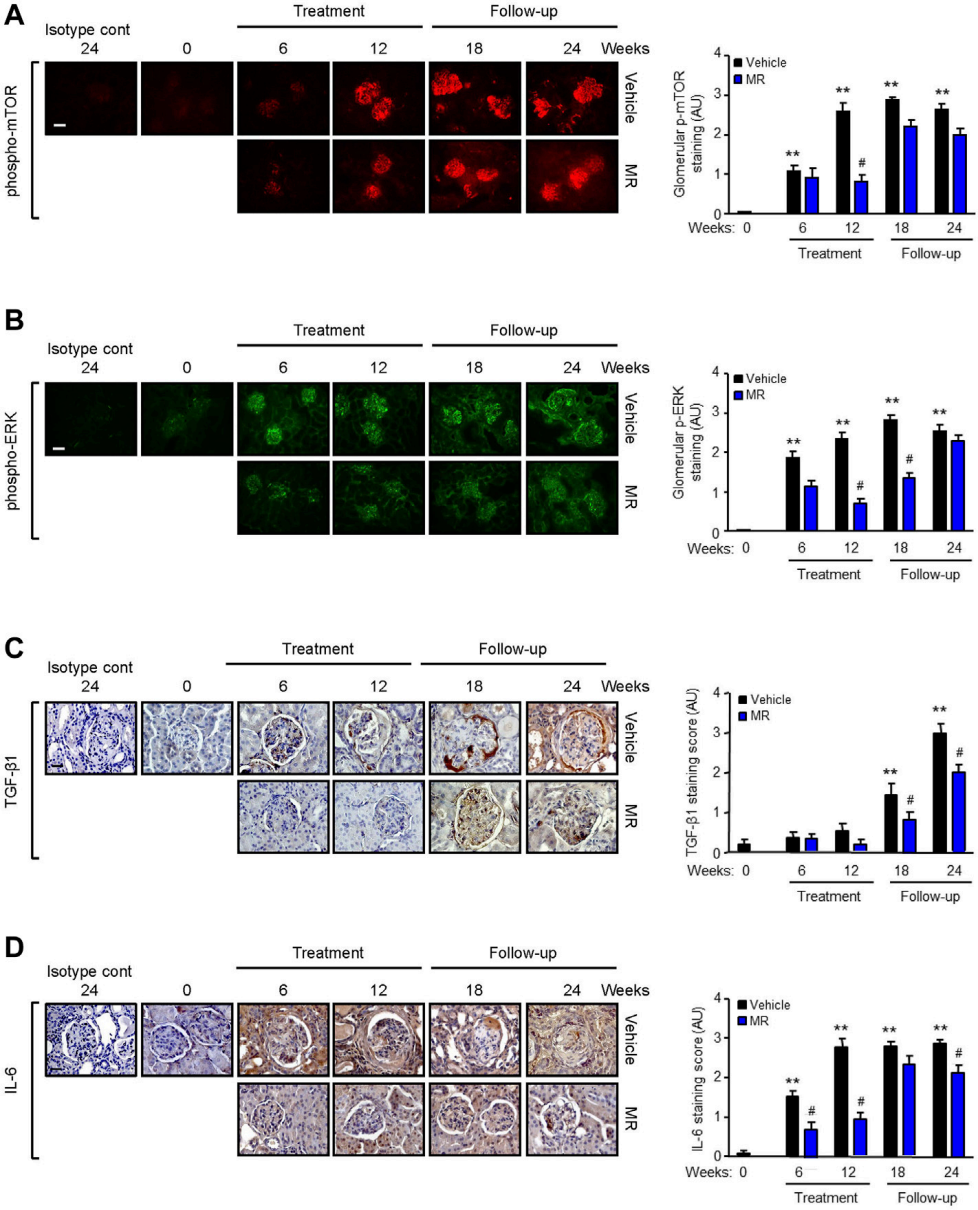
Effect of mycophenolate and rapamycin on mTOR and ERK phosphorylation and TGF-β1 and IL-6 expression in NZBWF1/J mice. Representative images showing **(A)** mTOR phosphorylation at Ser^2448^, **(B)** ERK phosphorylation, **(C)** TGF-β1, and **(D)** IL-6 expression in the kidneys of vehicle- and MR-treated mice at baseline (T = 0), 6, 12, 18, and 24 weeks. Isotype cont: isotype control. Original magnification x 200; Scale bar: 20 µm for panels A and B and original magnification x 400; Scale bar; 50 µm for panels C and D. mTOR and ERK phosphorylation and TGF-β1 and IL-6 expression were graded as described in the *Materials and Methods* and data expressed as mean ± SEM (right panels) (n = 6 mice per time-point per group). AU, arbitrary units. Data analyzed using repeated measures ANOVA with Bonferroni’s multiple comparison post-test. **p < 0.01, compared to baseline; ^#^ *p* < 0.05, vehicle vs. MR for the same time-point.

Since MR treatment showed comparable efficacy as that for monotherapy M or R in improving kidney histopathology, we next focused on the effect of combined MR on mediators of fibrosis. Increased mTOR phosphorylation at Ser^2448^, but not at Ser^2481^, and ERK phosphorylation were observed in the glomeruli of NZBWF1/J mice with active nephritis. As disease progressed, ERK phosphorylation was also detected in the tubulo-interstitium. Treatment with combined MR for 12 weeks resulted in marked reduction in mTOR and ERK phosphorylation, whereas activation of both signaling pathways increased when treatment was stopped (Figure 3A,B).

**FIGURE 4:**
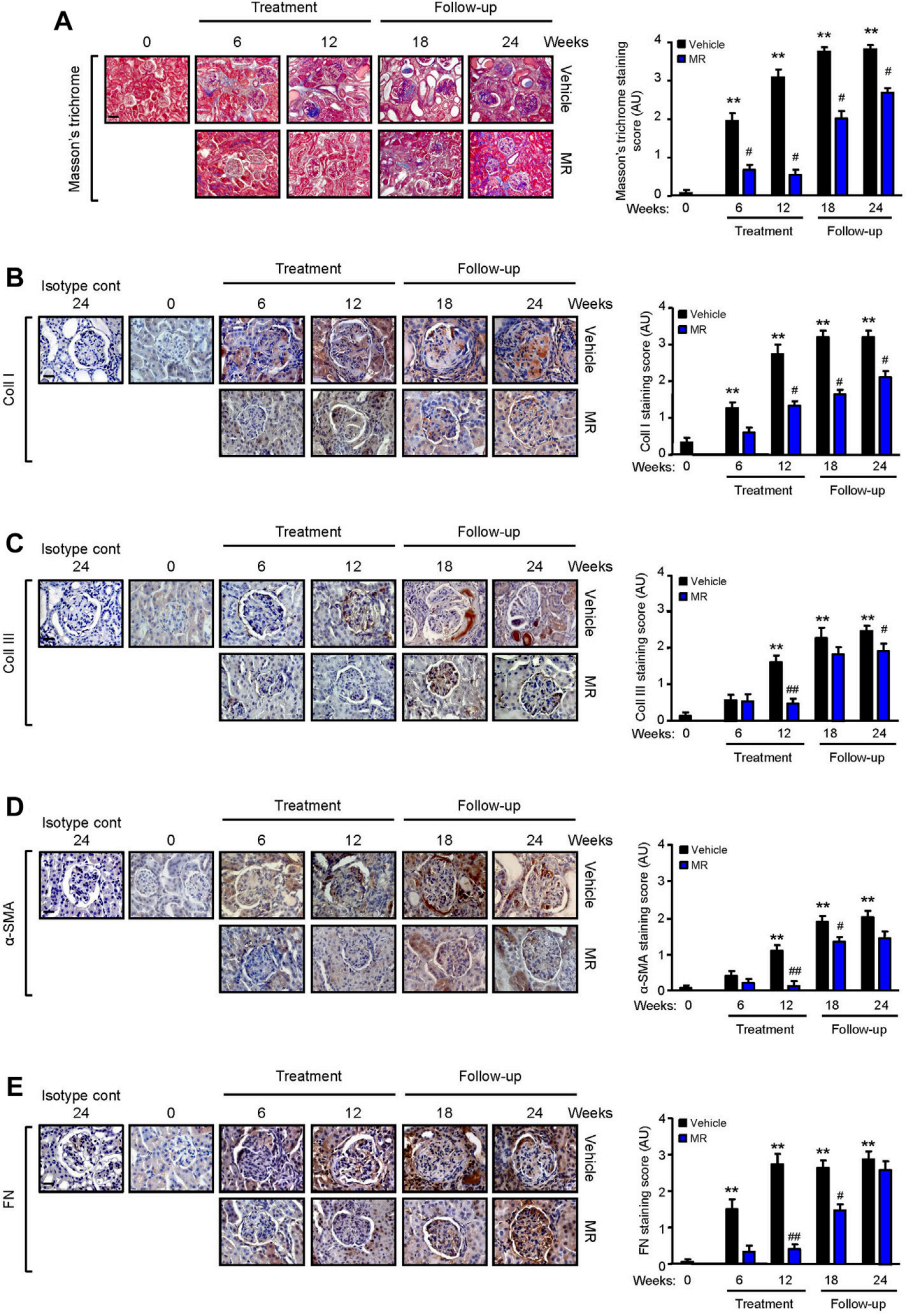
Effect of combined mycophenolate and rapamycin on collagen deposition, α-smooth muscle actin, and fibronectin expression in NZBWF1/J mice. Representative images showing **(A)** total collagen deposition as determined by Masson’s trichrome staining, **(B)** collagen I (Coll I), **(C)** collagen III (Coll III), **(D)** α-smooth muscle actin (α-SMA), and **(E)** fibronectin (FN) expression in the kidneys of vehicle- and MR-treated mice at baseline, 6, 12, 18, and 24 weeks. Original magnification x 200; Scale bar: 20 µm for panel A, and original magnification x 400; Scale bars 50 µm for panels B–E. Collagen, α-SMA, and FN expression in the kidneys of control and treated mice was graded as described in the *Materials and Methods*, and mean scores ±SEM are shown (n = 6 mice per time-point per group). AU, arbitrary units. Data analyzed using repeated measures ANOVA with Bonferroni’s multiple comparison post-test. **p < 0.01, compared to baseline; ^#^ *p* < 0.05; ^##^ *p* < 0.01, vehicle vs. MR for the same time-point.

TGF-β1 is a well-established mediator of kidney fibrosis, and long-term exposure to IL-6 has been reported to induce tissue fibrosis in unresolved inflammation (Yamamoto et al., 1993; Fielding et al., 2014). TGF-β1 expression was detected in the glomerulus and tubulo-interstitium after 18 and 24 weeks, respectively, and MR treatment reduced TGF-β1 expression (Figure 3C). Renal IL-6 expression increased progressively in control mice and was detected in the glomerulus and tubulo-interstitium after 6 weeks and in crescents and areas of interstitial fibrosis after 12 weeks. MR treatment significantly decreased glomerular and tubulo-interstitial IL-6 expression after 6 weeks of treatment, which was sustained for 12 weeks after cessation of treatment (Figure 3D). Increased collagen deposition, attributed at least in part to increased collagen I and III expression, was detected in the glomerulus and tubulo-interstitium of untreated mice at 6 weeks after commencement of the study, and this was accompanied by increased α-smooth muscle actin expression and fibronectin expression (Figure 4). Treatment with combined MR reduced these fibrotic markers after 6–12 weeks. Decreased α-smooth muscle actin and fibronectin expression was sustained for 6 weeks after cessation of treatment, whereas suppression of collagen I and III deposition was sustained to the cessation of the study (Figure 4).

### Effect of IL-6 and TGF-β1 on mTOR, PI3K, and ERK Phosphorylation and IL-6 Secretion in Human Mesangial Cells

**FIGURE 5:**
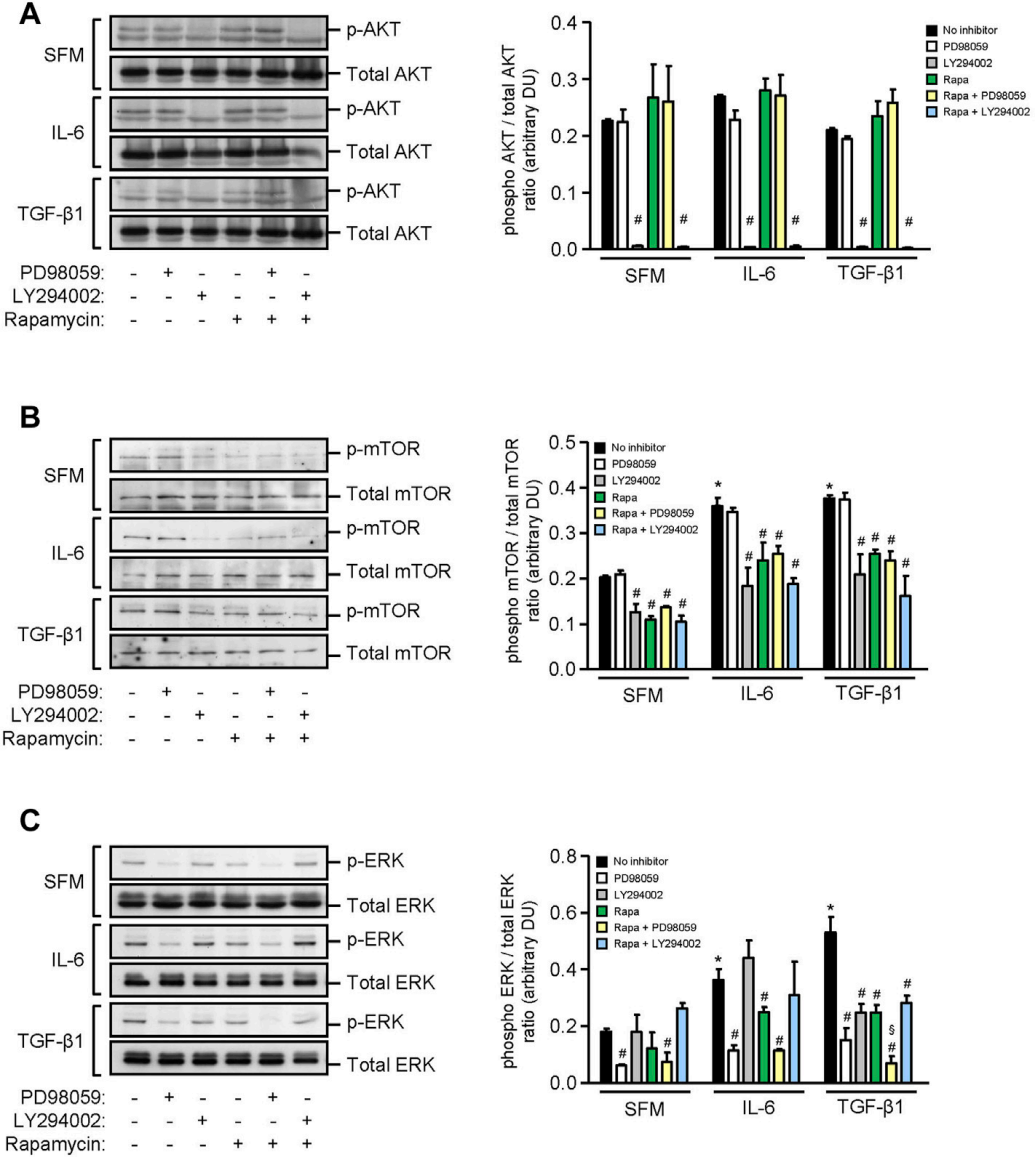
Effect of IL-6 or TGF-β1 on AKT, mTOR, and ERK phosphorylation in human mesangial cells. Representative Western blots showing the effect of SFM, IL-6, or TGF-β1 in the presence or absence of PD98059, LY294002, or rapamycin with or without PD98059 or LY294002 on **(A)** AKT phosphorylation, **(B)** mTOR phosphorylation at Ser^2448^, and **(C)** ERK phosphorylation, after 24 h (left panels). The intensity of each band was semi-quantitated using ImageJ, normalized to total AKT, total mTOR, and total ERK, and values expressed as mean ± SD for three separate experiments (right panels). DU, densitometric units. All data analyzed using ordinary ANOVA with Bonferroni’s multiple comparison post-test. *p < 0.05, SFM vs. IL-6 or TGF-β1; ^#^ *p* < 0.05, with vs. without inhibitor for the same stimulus; ^§^ *p* < 0.05, compared to rapamycin alone for the same stimulus.

We next investigated the signaling pathways that induced inflammatory and fibrotic mediators in HMCs. Under basal conditions, HMCs showed weak expression of AKT, mTOR, and ERK phosphorylation. Exogenous IL-6 increased mTOR and ERK phosphorylation by 1.80-fold and 2.01-fold, respectively, whereas TGF-β1 increased mTOR and ERK phosphorylation by 1.85-fold and 2.38-fold, respectively, in HMCs after 24 h stimulation. Neither IL-6 nor TGF-β1 had any effect on AKT phosphorylation after 24 h (Figure 5). Rapamycin and LY294002 (PI3K inhibitor) significantly decreased IL-6– and TGF-β1–induced mTOR phosphorylation, whereas PD98059 (ERK inhibitor) had no effect (Figure 5). PD98059, rapamycin, and LY294002 significantly decreased ERK phosphorylation that was induced by IL-6 or TGF-β1. Inhibition of ERK activation was more pronounced when combined rapamycin and PD98059 was used in cells stimulated with TGF-β1 (64.84, 42.44, and 83.73% reduction in cells incubated with PD98059, rapamycin, and combined rapamycin and PD98059, respectively) (Figure 5).

**FIGURE 6:**
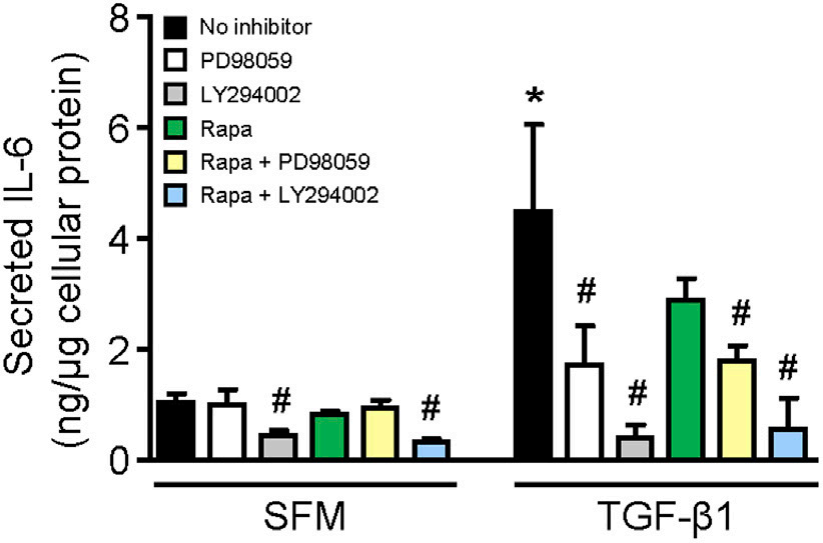
Effect of TGF-β1 on IL-6 secretion in human mesangial cells. Effect of TGF-β1 in the presence or absence of PD98059, LY294002, or rapamycin with or without PD98059 or LY29400 on IL-6 secretion in HMCs after 24-h incubation. Results are expressed as mean ± SD from three separate experiments. Data analyzed using ordinary ANOVA with Bonferroni’s multiple comparison post-test. *p < 0.05, SFM vs. TGF-β1; ^#^ *p* < 0.05, with vs. without inhibitor for the same stimulation.

Stimulation of HMCs with TGF-β1 for 24 h increased IL-6 secretion by 4.36-fold compared with that of cells incubated with SFM (*p* < 0.01, Figure 6). The results from experiments using specific inhibitors to ERK, mTOR, and PI3K showed that constitutive IL-6 secretion was mediated through PI3K since LY294002 reduced IL-6 secretion by 57.50%, whereas inhibition of ERK or mTOR had no effect (Figure 6). IL-6 secretion induced by TGF-β1 was mediated through ERK and PI3K since incubation of cells with PD98059 and LY294002 reduced IL-6 secretion by 61.72 and 91.14%, respectively (Figure 6). Rapamycin also decreased IL-6 secretion in HMCs stimulated with TGF-β1 (35.48% reduction), but it did not reach statistical significance.

### Effect of IL-6 and TGF-β1 on α-smooth Muscle Actin, Fibronectin, and Collagen III Expression in Human Mesangial Cells

**FIGURE 7:**
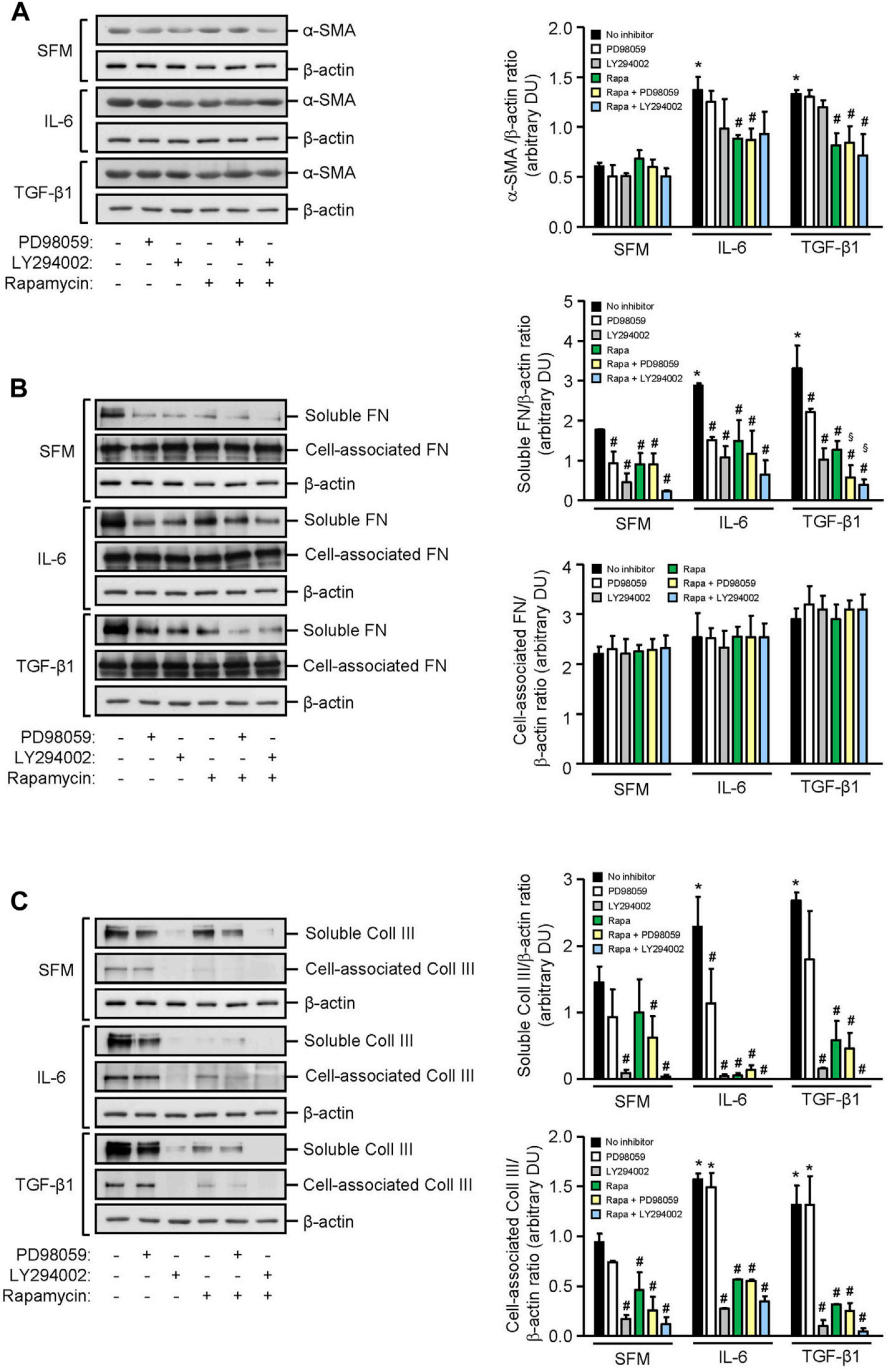
Effect of IL-6 or TGF-β1 on α-smooth muscle actin, fibronectin, and collagen III expression in human mesangial cells. Representative Western blots showing the effect of SFM, IL-6, or TGF-β1 in the presence or absence of PD98059, LY294002, or rapamycin with or without PD98059 or LY294002 on **(A)** α-smooth muscle actin (α-SMA), **(B)** soluble and cell-associated fibronectin (FN), and **(C)** soluble and cell-associated collagen III (Coll III) after 24 h (left panels). The intensity of each band was semi-quantitated using ImageJ, normalized to β-actin, and values expressed as mean ± SD for three separate experiments (right panels). DU, densitometric units. All data analyzed using ordinary ANOVA with Bonferroni’s multiple comparison post-test. *p < 0.05, SFM vs. IL-6 or TGF-β1; ^#^ *p* < 0.05, with vs. without inhibitor for the same stimulus; ^§^ *p* < 0.05, compared to rapamycin alone for the same stimulus.

HMCs constitutively expressed α-smooth muscle actin and soluble and cell-associated fibronectin and collagen III. IL-6 significantly increased α-smooth muscle actin, soluble fibronectin, and soluble and cell-associated collagen III expression after 24 h when compared to SFM. mTOR phosphorylation, but not PI3K or ERK phosphorylation, contributed to IL-6–induced α-smooth muscle actin since incubation of HMCs with rapamycin reduced α-smooth muscle actin expression by 35.51% (*p* < 0.05, Figure 7). Accumulation of fibronectin in the extracellular matrix is dependent on the activation and polymerization of soluble fibronectin monomers to form activated dimers (Wierzbicka-Patynowski and Schwarzbauer, 2003). Constitutive and IL-6–induced soluble fibronectin expression was mediated through PI3K, mTOR, and ERK phosphorylation since incubation with LY294002, rapamycin, and PD98059 significantly reduced soluble fibronectin expression, whereas PI3K, mTOR, and ERK phosphorylation had no apparent effect on cell-association fibronectin. Constitutive and IL-6–induced soluble and cell-associated collagen III were mediated through PI3K and mTOR phosphorylation. IL-6 induction of soluble but not cell-associated collagen III was also mediated through ERK phosphorylation (Figure 7).

TGF-β1 significantly increased α-smooth muscle actin, soluble fibronectin, and soluble and cell-associated collagen III expression in HMCs, and the increase of all three mediators of fibrosis was comparable to that observed with IL-6. Induction of α-smooth muscle actin by TGF-β1 was mediated, in part, through mTOR activation since incubation with rapamycin decreased α-smooth muscle actin by 38.65%. Induction of soluble fibronectin was mediated through PI3K, mTOR, and ERK phosphorylation since incubation with LY294002, rapamycin, and PD98059 decreased soluble fibronectin expression by 68.93, 61.62, and 42.15%, respectively. HMC co-incubated with rapamycin and PD98059 or rapamycin and LY294002 further reduced soluble fibronectin expression (82.19 and 88.53% reduction, respectively, Figure 7). Soluble and cell-associated collagen III induced by TGF-β1 was mediated through PI3K and mTOR phosphorylation since pre-incubation with LY294002 and rapamycin decreased soluble collagen III by 94.40 and 78.36%, respectively, and cell-associated collagen III by 92.28 and 75.85%, respectively (Figure 7).

### Effect of MPA and Rapamycin on Inflammatory and Fibrotic Processes Induced by IL-6 and TGF-β1 in Human Mesangial Cells

**FIGURE 8:**
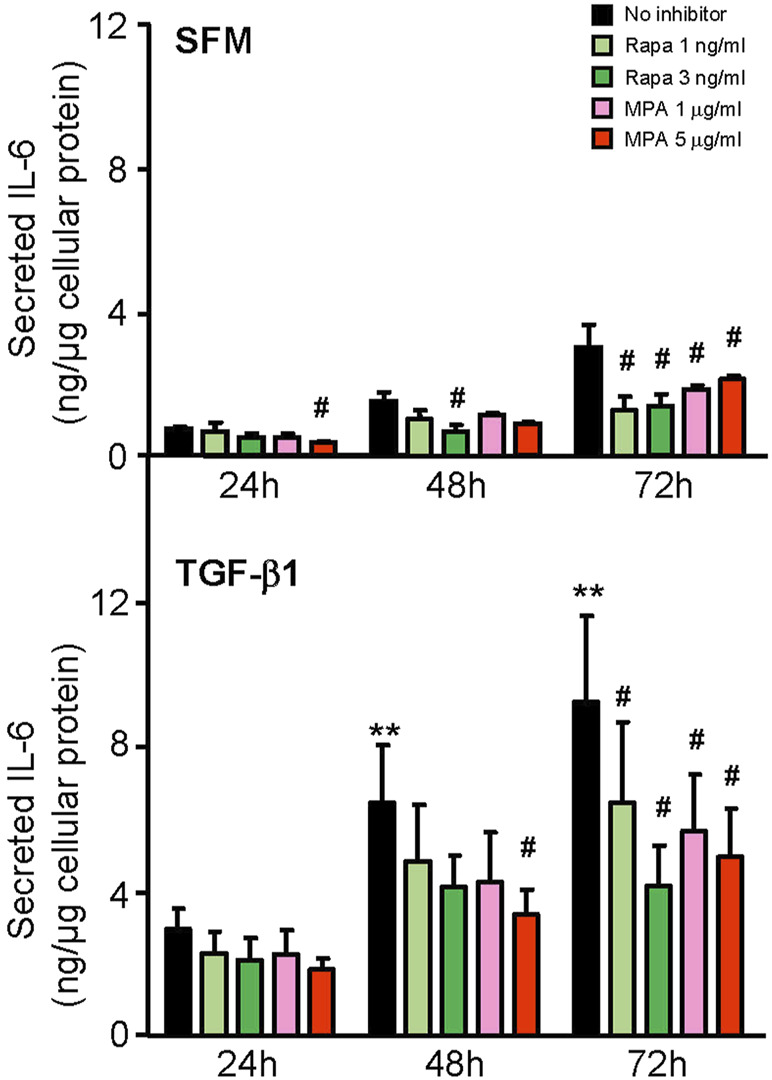
Effect of MPA or rapamycin on IL-6 secretion induced by TGF-β1 in human mesangial cells. Effect of SFM (upper panel) or TGF-β1 (lower panel) in the presence or absence of rapamycin (1 or 3 ng/ml) or MPA (1 or 5 μg/ml) on IL-6 secretion in HMCs. Results are expressed as mean ± SD from three separate experiments. Data analyzed using ordinary ANOVA with Bonferroni’s multiple comparison post-test. **p < 0.01, SFM vs. TGF-β1 for the same time-point; ^#^ *p* < 0.05, with vs. without rapamycin or MPA for the same stimulation and time-point.

We next compared the effect of MPA and rapamycin on mediators of inflammation and fibrosis that were induced by IL-6 and TGF-β1 in HMCs. MPA at 5 μg/ml decreased constitutive IL-6 secretion after 24 h and was sustained for 72 h (*p* < 0.05). Rapamycin at 3 ng/ml also reduced constitutive IL-6 secretion, but the inhibitory effect was not apparent until after 48 h and was sustained up to 72 h. TGF-β1 induced IL-6 secretion in a time-dependent manner. MPA reduced TGF-β1–induced IL-6 secretion after 48 h, whereas rapamycin decreased IL-6 secretion after 72 h (*p* < 0.05, for both) (Figure 8).

**FIGURE 9:**
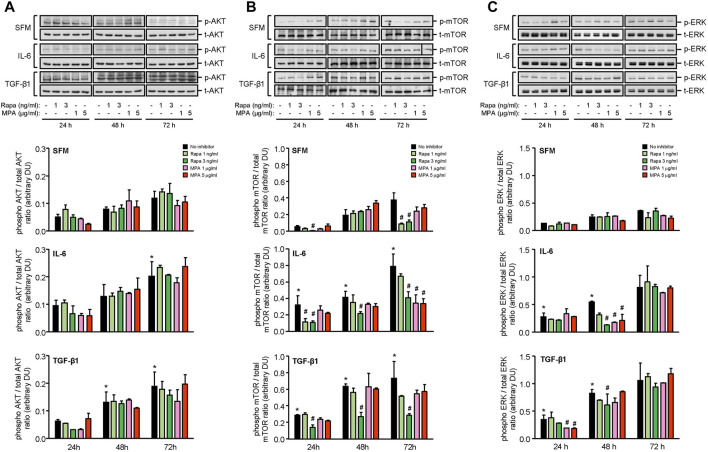
Effect of MPA or rapamycin on AKT, mTOR, and ERK phosphorylation in human mesangial cells.Representative Western blots showing the effect of SFM, IL-6, or TGF-β1 in the presence or absence of rapamycin (1 or 3 ng/ml) or MPA (1 or 5 μg/ml) on **(A)** AKT phosphorylation, **(B)** mTOR phosphorylation at Ser^2448^, and **(C)** ERK phosphorylation. The intensity of each band was semi-quantitated using ImageJ and normalized to total AKT, total mTOR, or total ERK. Values are expressed as mean ± SD for three separate experiments. DU, densitometric units. All data analyzed using ordinary ANOVA with Bonferroni’s multiple comparison post-test. *p < 0.05, SFM vs. IL-6 or TGF-β1; ^#^ *p* < 0.05, with vs. without rapamycin or MPA for the same stimulation and time-point.

**FIGURE 10:**
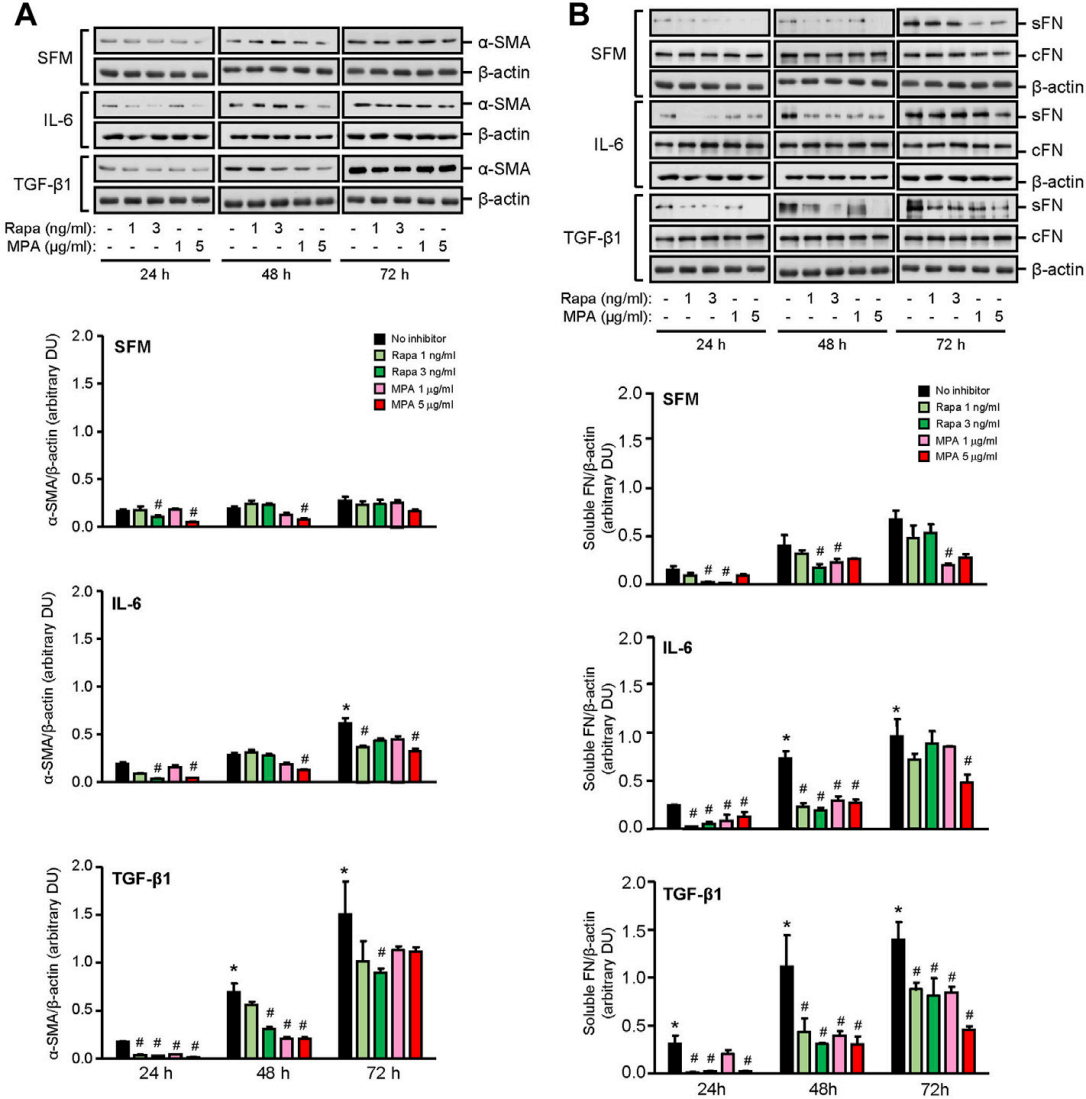
Effect of MPA or rapamycin on α-smooth muscle actin and fibronectin expression in human mesangial cells. Representative Western blots showing the effect of SFM, IL-6, or TGF-β1 in the presence or absence of rapamycin (1 or 3 ng/ml) or MPA (1 or 5 μg/ml) on **(A)** α-smooth muscle actin (α-SMA) and **(B)** soluble (sFN) and cell-associated FN (cFN). The intensity of each band for α-SMA and sFN was semi-quantitated using ImageJ and normalized to β-actin. Values are expressed as mean ± SD for three separate experiments. DU, densitometric units. All data analyzed using ordinary ANOVA with Bonferroni’s multiple comparison post-test. *p < 0.05, SFM vs. IL-6 or TGF-β1; ^#^ *p* < 0.05, with vs. without rapamycin or MPA for the same stimulation.

IL-6 and TGF-β1 induced AKT, mTOR, and ERK phosphorylation in a time-dependent manner. Rapamycin decreased IL-6- and TGF-β-induced mTOR phosphorylation after 24 h, which was sustained for 72 h. MPA decreased IL-6- but not TGF-β1-induced mTOR phosphorylation after 72 h (Figure 9B). MPA and rapamycin also reduced ERK phosphorylation that was induced by IL-6, whereas rapamycin but not MPA transiently decreased TGF-β1–induced ERK phosphorylation (Figure 9C). Both drugs had no effect on AKT phosphorylation (Figure 9A). IL-6 and TGF-β1 induced α-smooth muscle actin and soluble fibronectin in a time-dependent manner. Both MPA and rapamycin reduced IL-6– and TGF-β1–induced α-smooth muscle and soluble fibronectin actin after 24 h, and this reduction was sustained for 72 h (Figure 10).

## Discussion

The main objective of immunosuppressive treatment in patients with active lupus nephritis is to suppress acute inflammatory processes and prevent nephron loss (Chan, 2015; Parodis and Houssiau, 2022). Current treatment regimens for lupus nephritis comprise an induction phase aimed at inducing remission and a maintenance phase to prevent disease flares. Despite improvement in the management of lupus nephritis patients over the past 3 decades, a significant proportion of patients develop CKD and progress to ESRD (Maroz and Segal, 2013; Zhang et al., 2016; Parodis and Houssiau, 2022). There is, therefore, a need to develop new treatment strategies to prevent progressive kidney fibrosis and preserve long-term kidney function (Yap et al., 2012b).

Combination therapy is often used in the treatment of lupus nephritis with the aim of achieving efficacy, while reducing toxicity associated with individual drugs. We previously reported that mycophenolate at 100 mg/kg/day and rapamycin at 3 mg/kg/day showed comparable immunosuppressive effects and efficacy in preventing kidney function deterioration and fibrosis in murine lupus nephritis (Zhang et al., 2019). We extended our study to investigate the effect of combined mycophenolate and rapamycin on kidney fibrosis in active murine lupus nephritis. When used in combination, the dose of each immunosuppressive agent could be halved to provide the same level of immunosuppression as mycophenolate or rapamycin monotherapy, and combined MR treatment maintained the antifibrotic properties of the drugs. Peripheral white blood cell count was comparable between monotherapy and combination therapy, and none of the treatment groups showed signs of infection. The advantages of using mTOR inhibitors are that unlike calcineurin inhibitors, they do not possess nephrotoxicity and they also exert anti-neoplastic effect. While there had been concerns that mTOR inhibitors may induce proteinuria and onset of glomerulonephritis in some kidney transplant patients, this was not observed in our study.

Although treatment was initiated at the onset of albuminuria, renal histopathological changes were relatively modest at baseline. Progressive lupus nephritis was characterized by the production of anti-dsDNA antibodies, immune complex deposition in the glomerulus, mesangial expansion, glomerulosclerosis, increased tubulo-interstitial inflammatory cell infiltration, tubular atrophy, interstitial fibrosis, and deterioration of kidney function. mTOR and ERK phosphorylation increased with progressive disease and was predominantly localized to the glomerulus, and this was accompanied by induction of TGF-β1, IL-6, α-smooth muscle actin, fibronectin, and collagen I and III. Combined MR treatment improved kidney histology and fibrotic processes and phenotypic disease manifestations after 12 weeks and was sustained up to 6–12 weeks after cessation of treatment. Combined MR treatment showed comparable efficacy as that of monotherapy in improving kidney histology and fibrotic processes, with comparable reduction in mTOR and ERK phosphorylation and mediators of fibrosis. MR treatment reduced TGF-β1, IL-6, collagen I, collagen III, α-smooth muscle actin, and fibronectin expression. When treatment was stopped, the inhibitory effect of MR on α-smooth muscle actin and fibronectin expression was sustained for 6 weeks, whereas the inhibitory effect on TGF-β1, IL-6, and collagen I and collagen III expression persisted until the cessation of the study. It is possible that the sustained reduction in TGF-β1 and IL-6 expression may result in a concomitant downstream reduction in collagen expression, although further studies are warranted to confirm this, whereas the regulation of fibronectin and α-smooth muscle actin may be through additional mediators such as TNF-α or MCP-1 (Yung et al., 2015a), the levels of which may be increased following cessation of treatment.

TGF-β1 is a key mediator of kidney fibrosis, and IL-6 has been reported to drive tissue fibrosis in unresolved inflammation (Fielding et al., 2014). Increased IL-6, α-smooth muscle actin, fibronectin, and collagen I and III expression in the kidney was detected before TGF-β1 expression, suggesting that TGF-β1 may amplify fibrotic processes once fibrosis is established, but it does not appear to play a role in the initiation of kidney fibrosis in lupus nephritis. The role of TGF-β1 in fibrogenesis in lupus nephritis is controversial. Independent researchers have reported an increase in TGF-β1 expression, which was associated with increased fibronectin expression in renal specimens from patients and mice with active lupus nephritis (Yamamoto et al., 1994; Yamamoto et al., 1996; Yamamoto and Loskutoff, 2000), whereas other investigators suggested that kidney fibrosis in lupus nephritis patients occurred through proinflammatory mediators such as MCP-1 and not TGF-β1 since microarray analysis of glomeruli that were isolated from lupus nephritis patients showed reduced TGF-β1 expression, whereas MCP-1 expression clustered with genes related to fibrogenesis (Peterson et al., 2004).

Data from our *in vitro* studies demonstrated that induction of mTOR and ERK phosphorylation, α-smooth muscle actin, and mediators of fibrosis by IL-6 was comparable to that observed with TGF-β1, and it is plausible to suggest that IL-6 may contribute to kidney fibrosis in lupus nephritis. TGF-β1 was shown to increase IL-6 secretion in HMCs, which was mediated through increased PI3K, mTOR, and ERK phosphorylation and suggests that TGF-β1 may amplify the fibrotic effects of IL-6. We previously reported that IL-6 induced soluble fibronectin in proximal tubular epithelial cells (Yung et al., 2015a). In this study, we demonstrated that IL-6 induced α-smooth muscle actin, fibronectin, and collagen III in HMCs, and this underscores the contribution of proinflammatory mediators in kidney fibrosis. In line with our studies, Chen et al. reported on the role of IL-6 trans-signaling in murine models of kidney fibrosis (Chen et al., 2019). The importance of IL-6 in lupus nephritis pathogenesis is underscored in murine studies, whereby disruption of IL-6 signaling in lupus-prone mice was associated with reduced anti-dsDNA antibody production and proteinuria and improvement in kidney function (Kiberd, 1993; Finck et al., 1994). Mycophenolate has been shown to decrease IL-6 secretion in cultured proximal and distal tubular epithelial cells (Baer et al., 2004), whereas there are conflicting data on the effect of rapamycin on renal IL-6 expression. In a murine model of anti-GBM glomerulonephritis, mice treated with rapamycin at the time of immunization were protected from glomerulonephritis and renal IL-6 expression was reduced, whereas rapamycin treatment given 14 days after immunization resulted in a significant increase in both albuminuria and renal IL-6 expression, suggesting that the time when rapamycin treatment is initiated determines whether the drug exerts a beneficial or otherwise effect (Hochegger et al., 2008). In this study, we demonstrated that both MPA and rapamycin reduced IL-6 secretion in HMCs following TGF-β1 stimulation, although the anti-inflammatory/antifibrotic effect of MPA appeared earlier than that of rapamycin. In our animal studies, mycophenolate and rapamycin, whether administered as monotherapy or combination therapy to NZBWF1/J mice with active nephritis, showed comparable efficacy in suppressing IL-6 expression in the kidney (unpublished data). It is plausible that decreased IL-6 expression in the glomeruli and tubulo-interstitium following MR treatment was attributed to both immunosuppressive agents.

**FIGURE 11:**
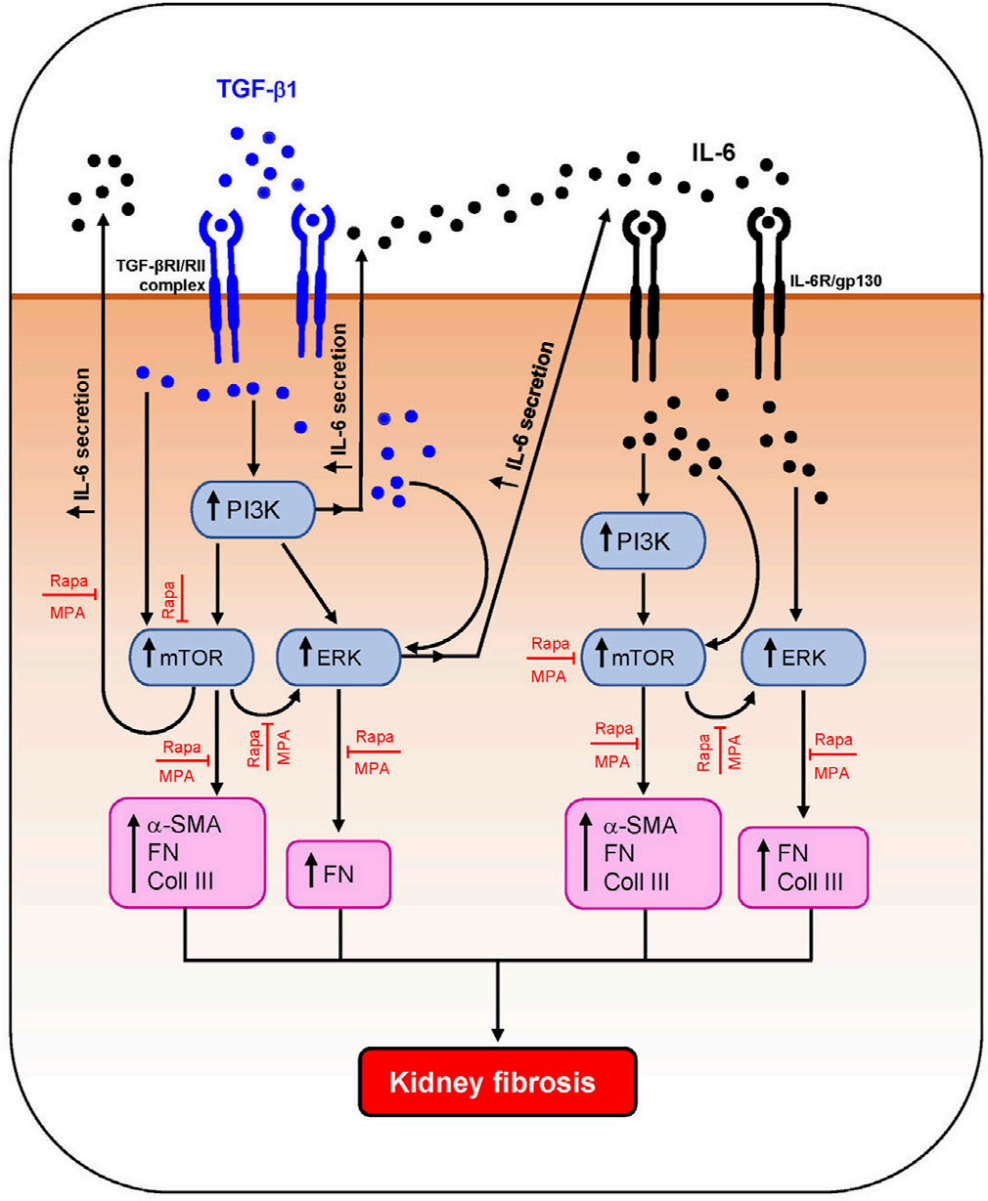
Schematic diagram summarizing the effect of MPA and rapamycin on TGF-β1– and IL-6–induced inflammatory and fibrotic processes in human mesangial cells. TGF-β1 induced AKT/PI3K, mTOR, and ERK phosphorylation and downstream IL-6 secretion and α-smooth muscle actin (α-SMA), collagen III (Coll III), and fibronectin (FN) expression in HMCs. IL-6 also induced the aforementioned signaling pathways and mediators of fibrosis in HMCs. Both mycophenolic acid (MPA) and rapamycin (Rapa) significantly decreased mTOR and ERK phosphorylation and downstream inflammatory and fibrotic processes but had no effect on AKT/PI3K phosphorylation.

Activation of PKC, TGF-β/SMAD, mTOR, and MAPK signaling pathways has been shown to contribute to kidney fibrosis (Yung et al., 2009; Yung et al., 2015a; Yung et al., 2017; Feng et al., 2018). IL-6 and TGF-β1 induced PI3K, mTOR, and ERK phosphorylation in HMCs, which was accompanied by downstream induction of IL-6 secretion and α-smooth muscle actin, fibronectin, and collagen III expression. IL-6 and TGF-β1 induced α-smooth muscle actin through mTOR phosphorylation. Induction of fibronectin was mediated through ERK, PI3K, and mTOR activation, and induction of collagen III was mediated through PI3K/mTOR signaling. Mycophenolate can decrease fibrotic processes in the kidney through downregulation of TGF-β1 expression and PKC, ERK, p38 MAPK, JNK, and mTOR activation (Yung et al., 2009; Yung et al., 2017; Zhang et al., 2019). In this study, mycophenolate and rapamycin decreased ERK phosphorylation that was induced by IL-6 and TGF-β1, and the inhibitory effect was comparable between both drugs. Mycophenolate and rapamycin also decreased mTOR activation, although the suppressive effect of mycophenolate on mTOR activation was less rapid and less effective than that of rapamycin. In animal models of CKD and tubulo-interstitial fibrosis, inhibition of ERK signaling using trametinib attenuated mTORC1 activation, suggesting that the ERK signaling pathway is upstream of mTORC1 (Andrikopoulos et al., 2019). In this study, we demonstrated that ERK phosphorylation was downstream of mTOR signaling in HMCs since inhibition of mTOR signaling with rapamycin resulted in a decrease in ERK phosphorylation, whereas PD98059 had no effect on mTOR phosphorylation. This discrepancy may be due to the cell-type, mediator that induced injury, disease model, and the time of sample collection. It is possible that mycophenolate reduced mTOR phosphorylation through downregulation of ERK phosphorylation, although further studies are necessary to confirm this. Neither mycophenolate nor rapamycin has any effect on PI3K phosphorylation, suggesting that PI3K activation is upstream of the actions of these immunosuppressive agents. Mycophenolate and rapamycin reduced α-smooth muscle actin expression in HMCs through inhibition of mTOR but not ERK phosphorylation, whereas suppression of fibronectin expression by both drugs was likely mediated through the inhibition of ERK and mTOR phosphorylation. Although mesangial cells are key mediators of kidney fibrosis, other resident renal cells and immune cells such as proximal tubular epithelial cells, fibroblasts/myofibroblasts, and macrophages also contribute to kidney fibrosis. We and others have shown that mycophenolate and rapamycin can exert anti-inflammatory and antifibrotic effects on proximal tubular epithelial cells and fibroblasts, which may also contribute to the improvement in kidney structure and fibrotic processes (Badid et al., 2000; Copeland et al., 2007; Yung et al., 2015a; Yung et al., 2017). Figure 11 summarizes the effect of MPA and rapamycin on signaling pathways and inflammatory and fibrotic processes induced by TGF-β1 and IL-6 in HMCs. Our findings demonstrated that both MPA and rapamycin can exert direct anti-inflammatory and antifibrotic effects in HMCs.

Our animal studies demonstrated that treatment of NZBWF1/J mice with combined mycophenolate and rapamycin at half the doses used in monotherapy improved the structural integrity of the kidney and prevented deterioration of kidney function. Both immunosuppressive agents exerted their antifibrotic effects directly on mesangial cells. Our clinical evidence suggests efficacy and safety of using mTOR inhibitors in the treatment of lupus nephritis patients (Yap et al., 2012a; Yap et al., 2018). Based on the findings from our translational and clinical studies, further studies may be warranted to investigate the combined use of mycophenolate and rapamycin in the clinical management of lupus nephritis, which would be helpful when tailoring treatment according to the specific characteristics of each patient with the objective of maximizing the benefits of the medication, while minimizing the side effects of each drug.

In conclusion, data from our animal studies demonstrated that combined mycophenolate and rapamycin reduced kidney fibrosis and improved kidney function. The suppressive effect on fibrotic processes in HMCs suggests a direct effect on resident kidney cells that was most likely independent of their immunosuppressive actions. The antifibrotic effects of mycophenolate and rapamycin were mediated, at least in part, through their ability to inhibit mTOR and ERK phosphorylation. We also demonstrated the importance of proinflammatory mediators in kidney fibrosis and the contribution of IL-6 in inducing fibrogenesis in lupus nephritis.

## Abbreviations

ACR, albumin-to-creatinine ratio; AU, arbitrary units; Coll I, collagen I; collagen III, collagen III; DU, densitometric unit; ECL, enhanced chemiluminescence; ELISA, enzyme-linked immunosorbent assay; ERK, extracellular signal–regulated kinase; FBS, fetal bovine serum; FN, fibronectin; H&E, hematoxylin and eosin; HMCs, human mesangial cells; IgG, immunoglobulin G; IL-6, interleukin-6; mTOR, mammalian or mechanistic target of rapamycin; MCP-1, monocyte chemoattractant protein-1; NZBWF1/J, New Zealand Black and White first generation; PKC, protein kinase C; PI3K, phosphoinositide 3-kinase; SFM, serum-free medium; SLE, systemic lupus erythematosus; α-SMA, α-smooth muscle actin; TGF-β1, transforming growth factor beta1; TNF-α, tumor necrosis factor-alpha.

## Data Availability

The original contributions presented in the study are included in the article/Supplementary Material; further inquiries can be directed to the corresponding authors.
